# Association between Grain Intake, Nutrient Intake, and Diet Quality of Canadians: Evidence from the Canadian Community Health Survey–Nutrition 2015

**DOI:** 10.3390/nu11081937

**Published:** 2019-08-17

**Authors:** Seyed H Hosseini, Julie M Jones, Hassan Vatanparast

**Affiliations:** 1 College of Pharmacy and Nutrition, University of Saskatchewan, Saskatoon, SK S7N 2Z4, Canada; 2 Professor Emerita of Foods Nutrition, St. Catherine University, St. Paul, MN 55105, USA

**Keywords:** whole grains, balanced intakes of grain, propensity score matching, obesity

## Abstract

The new Canada’s Food Guide (CFG) recommends whole grains foods as the primary choice of grain products in the daily diet. This study examined whether higher shares of whole-grain consumption, beyond the recommended levels (i.e., above half) of the daily grain intake, are linked with optimal diet quality and intakes of some key nutrients, for both children and adolescents and adults in Canada. To meet the objective of this study, we used the Canadian Community Health Survey (CCHS)–Nutrition 2015, which is a nationally representative data. We employed the propensity score matching (PSM) method in this study. PSM estimates the exposure effect when a set of individuals are exposed to a specific treatment (food group intake in this study) in a non-experimental setting. The results of our analyses implied that a high consumption of whole grains is associated with a good diet quality. However, after a certain level of whole-grain consumption, no significant differences can be observed in diet quality scores of children and adolescents and adults. Moreover, it was observed that the proportion of obese and overweight individuals was significantly lower among adults that had balanced intakes of whole and non-whole grains. The results of logistic regression analyses also showed the probability of being obese and overweight is significantly lower in the case of adults with balanced intakes of grains. However, no significant differences were observed in the prevalence of obesity and overweight across whole grains consumption patterns for children and adolescents.

## 1. Introduction

Grain products are one of the main staple foods across the globe. Data from the 2004 and 2015 Canadian Community Health Survey (CCHS) showed that grain-based foods (GBFs) are important sources of folate, thiamine, iron, fiber, and energy in the daily diet [1]. On average, these foods contributed 45% of folate, 41% of iron, 35% of fiber, and 25.9% of energy per day to the Canadian diet. The new Canada’s Food Guide (CFG) released in January 2019 emphasizes whole grains and suggests that they should be substituted for refined grains. Grains include the germ, the endosperm, and the bran. Therefore, whole grain foods are defined as those products that include all three parts of a grain. In contrast, the refined grain products are those foods that do not include the germ and the bran [2]. The previous CFG recommended that at least half of the grain products consumed daily should be whole grains [3]. These are based on the observations linking whole grain intake to a lowered risk of cardiovascular diseases and a reduced body weight [4,5,6,7]. Consumption of whole grain foods is associated with a lower risk of developing colorectal cancer [8], type 2 diabetes [9,10], cancer, and mortality [11,12]. Furthermore, previous research suggests consuming higher amounts of refined grains is linked with higher frequencies of type 2 diabetes [13] and the metabolic syndrome [14]. Thus, recommendations are made toward substituting refined grains with whole grains in the daily diet [8,12].

Whole grains contribute key nutrients to our diet, including dietary fiber, protein, vitamin B6, and magnesium [15]. Additionally, the mandatory fortification policy of grains in Canada has made refined grains a source of a few vital nutrients [16]. According to the Canadian Food Inspection Agency (2017), flour (i.e., white or refined flour) is required to be fortified with iron, folic acid, riboflavin, thiamine, and niacin. Furthermore, vitamin B6, magnesium, d-pantothenic acid, and calcium could be added to flour voluntarily [16]. Among the nutrients added to refined grains, iron and calcium are the nutrients of public health concern in Canada [17]. Thus, voluntary and mandatory fortification of refined grains contributes to the intakes of these important nutrients. Given the contribution of both whole and refined grains in the provision of important nutrients, the objective of this study was to examine whether a diet including mostly whole grain provides an optimal intake of nutrients and diet quality (defined by Nutrient Rich Food index) in Canada. 

## 2. Materials and Methods

### 2.1. Data

The CCHS–Nutrition 2015 data was used in this study. CCHS–Nutrition 2015 is a cross-sectional survey including more than 20,000 individuals that were representative of Canada’s population. The CCHS–Nutrition 2015 includes Canadians aged one year and older who are living in private dwellings in all provinces of Canada. Those who live in institutions, military bases, and indigenous reserves are not included in the survey. In our study, following Barr et al. (2018), we excluded pregnant women, breastfeeding women, and those with implausible energy intakes of below 200 Kcal or above 8000 Kcal [18]. 

The survey collected different types of information, including, but not limited to, participants’ dietary intake and socioeconomic status (SES). The dietary intake data were collected in two inconsecutive 24 h recall interviews using an automated multiple pass method [19]. About one-third of the participants were interviewed on a different day of the week for the sake of estimating the usual intakes of nutrients and foods. In this study, we used the information gathered from the first interview to conduct the analyses. Using the Tiers classification of Health Canada [20], three types of grain-based foods were considered in this study, including whole grain, non-whole-grain–enriched, and non-whole-grain–not-enriched. The term enriched in here refers to the presence of mandatory fortification of refined flour in Canada [16]. 

### 2.2. Method

To measure differences in nutrient intake and diet quality across whole-grain consumption patterns (WGCs), we used the PSM. This method estimates the treatment effect when a set of individuals are exposed to a specific treatment in a non-experimental situation. PSM intends to estimate the non-biased impact of a treatment when there is no systematic way to distinguish between the treatment and control groups [21]. In non-technical terms, PSM uses data from control groups to detect what would have happened to the treated groups if they had not been exposed to the treatment [22]. Matching methods are employed to pair the control and treatment groups based on their observable characteristics. However, the important question is—on the basis of which characteristic(s) should a researcher match the treated and untreated groups? In this situation, PSM suggests the use of a score-matching method reflecting all characteristics of individuals in one dimension [21].

In this study, we considered the patterns of whole-grain consumption to examine the differences in diet quality in terms of Nutrient Rich Food index version 9.3 (NRF 9.3) and nutrient intakes for two age groups of 2- to 18-years-old and 19-years-old and over. In this study, we distinguished between five patterns of whole-grain consumption plus a ‘minimal grain’ consumption pattern. These patterns were as follows—the lowest, where the share of whole grains from the total daily-grain-intake was between 0% to 20%; low, where the share of whole grains from the total daily-grain-intake was between 20% to 40%; balanced, where the share of whole grains from the total daily-grain-intake was between 40% to 60%; high, where the share of whole grains from the total daily-grain-intake was between 60% to 80%; the highest, where the share of whole grains from the total daily-grain-intake was above 80%; and finally ‘minimal grain’ consumers, where the total daily intake from any grain was less than one serving per day. The primary reason behind considering these categories was the recommendations made by the CFG and the United States Department of Agriculture, suggesting that at least half of the grain intakes should be whole grains [3,23]. Following these recommendations and the previous studies showing that refined grains could contribute to the intakes of some key nutrients for both children and adults in Canada [24,25], a pattern of whole-grain consumption (i.e., the balanced intake of grains) was defined where the share of whole grain intakes was between 40% to 60% of the total daily intakes of grain. The other patterns of whole grain intakes in this study were defined around the balanced pattern of consumption 

The measure of diet quality in our study was NRF 9.3 [26]. NRF 9.3 measures the quality of diet in terms of the intakes from 12 nutrients based on their recommended daily value. This measure assigns positive weights to the intakes of nine nutrients—fiber, protein, vitamin A, vitamin C, vitamin E, vitamin B-12, calcium, iron, magnesium, zinc, and potassium. However, three nutrients, including sugar, sodium, and saturated fatty acids, are given negative weights if the intakes exceed the recommended daily values. 

In the case of adults (19 years and older), the variables used in the PSM for the matching were age, age squared, sex, immigration status, ethnicity (white, non-white), household education (a member of the household has a higher education degree), household income level (income decile), location of residence (urban versus rural), smoking behavior (smoker versus non-smokers), marital status (married versus non-married), and finally the province of residence. For children and adolescents, we used similar variables in the PSM estimation, but omitted ‘smoking’ and ‘marital status’. “TEFFECTS PSMATCH” in Stata [27] was used to conduct the PSM analyses for reporting the “average treatment effects on treated” measures. The postestimation analyses of “OVERLAP” and “TEBALANCE” were employed to check for the models’ behaviors in terms of providing reliable results. In estimating the PSM models, we assumed that all individual received the dietary exposure of interest with a positive probability. The “OVERLAP” test plots the probability density of receiving the treatment for a model estimated. We used the “OVERLAP” to test whether there was a considerable mass around zero and one implying that the overlap assumption was violated [27]. However, the mass around zero and one was not considered in any of the estimated models. “TEBALANCE” was a postestimation test, after PSM. The statistics reported by “TEBALANCE” indicated whether the distribution of a covariate changed over the levels of the dietary exposure (i.e., treatment). If such differences were observed, the balanced assumption was not satisfied [27]. 

We also compared the characteristics of the participants across different patterns of whole-grain consumption. These characteristics included age, body mass index (BMI), sex, immigration status, marriage status, ethnicity, income levels, food security status, smoking behaviors, obesity, and supplement intakes. Individuals were considered to be immigrants if they had landed in Canada as immigrants. Food security status was based on “income-related” food security status. The binary variable ‘smoker’ flagged those who currently smoke daily and occasionally. Body mass index (BMI) defined as the weight in kilogram divided by the squared value of height in centimeter. We used the BMI variable to derive the overweight/obesity variable, where a person was considered to be obese/overweight if her/his BMI was greater than 25 and non-obese/not-overweight otherwise. A binary variable named ‘active’ was also defined to distinguish between active and less-active respondents. A person was considered as active if she/he had 150 min or more of vigorous-to-moderate physical activity per week. 

Following the guidelines provided by Statistics Canada, and due to the complex survey design, the descriptive statistics were weighted and bootstrapped for attaining population-level outcomes. Bootstrapping is a process by which several samples are randomly chosen from a dataset (500 times in our case) and each time the specified model is estimated. The coefficients of each estimation are stored, and the standard deviation of the estimated coefficients are calculated and reported as the standard errors of the coefficients. The statistical differences of nutrient intakes and SES across patterns of consumption were identified using the overlaps between the 95% confidence intervals of the estimates [28]. The individuals who had less than one serving of any GBFs were categorized as ‘minimal grain’ consumers, treated as a separate dietary pattern along with the five WGCs.

To investigate the association between the patterns of whole-grain consumption and the prevalence of obesity and overweight, logistic regression was used in Section 3.3. The binary dependent variable in the regression model was equal to one if a person was obese or overweight and zero otherwise. In the model, we controlled for the intakes of fruits and vegetables, milk and alternatives (e.g., fluid milk, butter cheeses, yogurt, etc.), meats and alternatives (e.g., beef, poultry, fish, nuts, legumes, eggs, etc.), other protein foods, energy consumption, physical activity, smoking status, and a set of SES.

## 3. Results

Table 1 shows the percentage of children, adolescents, and adults in each WGCs. Over 63% of children and adolescents, and about 60% of adults were located in the lowest WGCs, meaning whole grains contributed less than 20% of their daily total grain intake. The proportion of children and adolescents and adults with the balanced intake of grains were roughly the same (8.5% and 8.8%), while the percentage of adults with the highest whole grain intake (i.e., above 80% of their grains as whole grains) was higher than that for children and adolescents. Adults also had a higher percentage of ‘minimal grain’ consumers (i.e., less than one serving of grain per day) than children and adolescents. 

### 3.1. Socioeconomic Status across Whole-grain consumption Patterns

Table 2 and Table 3 gives measures of SES, body weight, and lifestyle factors of adults and children across WGCs. Within the patterns of grain consumption, slightly more males than females were in the ‘lowest’ (51.2%) and the ‘low’ groups (53.8%) of whole-grain consumption patterns, and fewer males (45.3%) than females were in the ‘balanced’-intake of grains consumption pattern. There was nearly twice the percentage of smokers among the ‘minimal grain’ consumers and the lowest whole grain consumers in comparison with other WGCs categories. The prevalence of obese and overweight individuals was significantly higher among all dietary patterns of whole grain, compared to the balanced intakes of grain. Furthermore, the average BMI of adults in the balanced consumption pattern was significantly lower than those with the lowest, high, and the highest WGCs. For children and adolescents, there were no differences in the BMI Z-scores for any WGCs category, including ‘minimal grain’ (Table 3). Table 3 also illustrates that significantly higher proportions of children and adolescents living in food insecure and less educated households were in the lowest WGCs category, compared to those in the category eating a balanced intake of grains. 

### 3.2. Diet Quality, Intakes of Energy, and Nutrients across WGCs

Average treatment effects on the treated (ATE) are the average differences in diet quality, energy, and nutrients intakes of individuals having a specific pattern of grain consumption in comparison with all other patterns of grain consumption (Table 4 and Table 5). Thus, a negative ATE in the tables indicates that a dietary pattern is associated with a decrease in the particular nutrient or dietary component compared to all other patterns of whole-grain consumption. Mean NRF 9.3 score, energy, and nutrients intakes (unadjusted) of both age groups across WGCs are also reported in Table 4 and Table 5.

The NRF score (diet quality) increased steadily across the whole grain categories. Adults with the lowest WGCs had the lowest mean NRF 9.3 diet quality score (491.1). This score was significantly lower than the NRF score observed for all other WGCs and for the ‘minimal grain’ consumers. The mean NRF score for those in the lowest WGCs category was 71 points lower than for those in the highest WGCs category and 24 points lower than the ‘minimal grain ‘category. However, the mean NRF scores for the balanced WGCs category and the two higher categories were not significantly different and varied by 11 points. In other words, the intake of whole grains at levels above the ‘make at least half your grains whole’ recommendation did not improve the diet quality scores.

Table 5 presents information on average treatment effects on the treated and mean values of diet quality, energy, and nutrients intake in children and adolescents, for children and adolescents. The mean diet quality scores of the ‘minimal grain’ consumers (NRF = 463) and those in the lowest WGCs (NRF = 496) were significantly lower than all other groups. These scores were more than 50 points lower than for other WGCs categories. Thus, intake of more whole grain was associated with higher levels of diet quality. The mean levels of diet quality in low and the balanced whole grain diets were 549.6 and 564.3 points. Overall, children and adolescents with the highest share of whole grains in their daily grain intakes had a significantly higher diet quality score that equaled to 61.8, in comparison to the ATE of other WGCs. 

For both children and adults, energy intakes were significantly lower for the ‘minimal grain’ consumers than all other WGCs categories. The average intakes of energy for adults with a ‘minimal grain’ dietary pattern was 1456 kcal, and the figure for children and adolescents was 1252 kcal. In terms of the ATE measure, on average, the energy intakes of the ‘minimal grain’ adults consumers were 513 kcal lower than all consumption patterns of whole grains. Children and adolescents who consumed less than one serving of GBFs on average had 546 kcal lower intakes of energy on a daily basis. The average intakes of energy were lower across the higher shares of whole grain intakes in both age groups as well.

A comparable trend in the pattern of carbohydrate intakes was observed across WGCs for both age groups. Adults, children, and adolescents with ‘minimal grain’ consumption patterns had considerably lower intakes of carbohydrates. On average, the carbohydrate intakes of adults, children, and adolescents with ‘minimal grain’ dietary patterns were 142.7 g and 154.2 g, respectively, which were significantly different from each other. Considering the ATE of carbohydrate intakes in the case of adults and children and adolescents with a ‘minimal grain’ dietary pattern, lower intakes of carbohydrates were observed (−83 g for adults and −88 g for children and adolescents in comparison with other patterns of whole-grain consumption). 

Four vitamins and one mineral—folate, thiamin, riboflavin, niacin, and iron,—are mandatory additions to refined grain under Health Canada’s grain fortification policy. Mean folate levels were lowest for the ‘minimal grain’ consumers. With each increase in the WGCs category, the mean intake of folate acid decreased. Considering the PSM analyses, the higher levels of whole grains were associated with lower intakes of folate. In fact, folate intakes of individuals with the highest WGCs were significantly lower than the lowest, low, balanced, and high whole-grain consumption patterns.

Our analyses indicated that the intakes of folate, thiamin, riboflavin, niacin, iron, and calcium were lower in the case of individuals who had high and the highest share of whole grain intakes. Among these nutrients, iron and calcium are the nutrients of public health concerns [17]. However, the opposite is true for magnesium, potassium, and fiber that can be found mostly in whole grain foods, among which potassium is a nutrient of public health concern in Canada [17]. Calcium and magnesium, and B6 and pantothenic acid can also be added. 

The average iron intakes of adults in ‘minimal grain’ and the highest patterns of whole grain intakes were almost identical (8.3 mcg) among adults. However, when comparing the ATE across these two patterns, it was observed that the ‘minimal grain’ consumers had significantly lower intakes of iron in both age groups. PSM analyses showed the low and the balanced intakes of whole grain were associated with higher intakes of calcium, and compared to iron intakes, ‘minimal grain’ consumers had significantly lower intakes of calcium across both age groups (−214 mg for adults and −327.3 mg for children in comparison with other patterns of whole-grain consumption). 

Individuals with the lowest and low intakes of whole grain had significantly lower intakes of magnesium, potassium, and fiber. Comparing the ATE of fiber intakes across WGCs, no significant differences in the intakes of fiber were observed where the whole grain intake exceeded the balanced intakes of grain in adults. Similar trends were observed in the case of magnesium and potassium, in both age groups.

### 3.3. The Balanced Intake of Grains and the Prevalence of Obesity

As was observed, the percentage of obese and overweight adults was significantly lower in individuals with a balanced intake of grains than those observed in any other WGCs (Table 2). For further investigation, we conducted a logistic regression where the dependent variable was equal to one if a person was obese or overweight. Considering the balanced intake of grain as the reference group, it was observed that the odd ratios of all other WGCs (except the low pattern of whole-grain consumption) were significantly different from zero. 

As indicated in Table 6, the odds ratio of the ‘lowest’, ‘high’, and the ‘highest’ groups of whole-grain consumption patterns were 1.5, 1.8, and 1.6, respectively. The odds ratio of ‘minimal grain’ consumers with respect to a balanced intake of grains was 1.5. However, the coefficient of ‘minimal grain’ consumers was significant at the 10% level. In other words, the hazard risk of being obese or overweight increased if whole grain intake was higher or lower than a balanced intake. 

## 4. Discussion

In this study, we examined whether higher shares of whole-grain consumption, beyond 60% of daily grain intake, are associated with optimal intakes of some key nutrients and diet quality, in children, adolescents, and adults in Canada. Using, the CCHS–Nutrition 2015 and employing the PSM method, our analyses suggest that higher levels of diet quality are associated with increases in intakes of whole grains, up to a certain level. With higher intakes of whole grains, no significant differences could be observed in the diet quality scores of adults, children, and adolescents. Furthermore, the prevalence of obesity was significantly lower among adults with balanced intakes of whole and non-whole grains.

Overall, 70% of Canadians fail to meet the former grain recommendation to make ‘half their grains whole’. A study conducted in Australia showed that 73% of adults do not meet the recommended level of whole grain [29].

However, dietary guidelines for Americans [30] and the new CFG [31] released in January 2019 emphasize the consumption of more whole grains as “nutrient-rich” foods in comparison with refined grains. Specifically, new CFG recommends most of the daily grain intakes of individuals to be whole grains [31]. Thus, given the fact that most Canadians are in the lowest and low category of whole grains intake, recommendations that promote increased whole grain intake for the nearly three-fourths of the population whose intakes are below the balanced intake might be helpful. However, consuming too much whole grain relative to the fortified refined grains might not be advisable for optimal nutrition or food acceptance. Whole grain foods are better accepted, especially by children, when they have a mix of whole and refined grains [32,33].

Previous studies focusing on the association between whole-grain consumption and diet quality have also shown that an increase in consumption of whole grains in terms of the number of servings is associated with higher levels of diet quality in adults [34] and children and adolescents [35]. However, in epidemiological studies, the highest intakes are observed with three servings per day. Intervention studies with higher intakes have shown mixed results in the outcomes [36]. 

The fortification of grain products with important nutrients such as iron, folate, niacin, and calcium raises the question of whether higher shares of whole grain intakes are associated with optimal diet quality. Studies show that fortified foods are important sources of some nutrients [37,38,39]. Berner et al. (2014) indicated that in the absence of current GBFs’ fortification in the U.S, the percentage of children and adolescents, especially adolescent girls, with below Estimated Average Requirement (EAR) intakes of some nutrients would have considerably increased in the U.S. However, the authors discuss that refined grain are the major contributors of energy and sugar intakes among children and adolescents, in the U.S as well. Therefore, they recommend that the intakes of higher levels of whole grain should be accompanied by fortification of whole-grain products [38]. Similar Canadian data showed that fortification of GBFs resulted in marked reductions in inadequate intakes of vitamin A, vitamin C, magnesium, and folate, and improvements in calcium intakes for some age/sex groups (33,36). 

Balanced intakes of enriched, refined, and whole grain products are associated with optimal diet quality and intakes of some key nutrients. While some researchers suggest fortification of whole grain and refined grains , there are some limitations in such fortification, including laws and regulations on the addition of nutrients to foods as per the national standards, and regulations regarding the addition of nutrients to certain foods.

### Balanced Intakes of Grains and Obesity

We observed that the proportion of adults who are obese or overweight is significantly lower in the balanced intake of GBFs group (i.e., 40% to 60% whole-grain intake). These results were confirmed by logistic regression analyses adjusting for all potential confounders as well. Jacobs Intervention studies have not shown consistent results with respect to weight by substituting all whole grains [40,41]. In addition, a systematic review conducted by Pol et al. (2013) showed that the intake of whole grain does not have a significant impact on the body weight, and the consumption of this food group slightly affects the proportion of body fat in healthy adults [42].

Merchant et al. (2009) examined the association between obesity and carbohydrate intakes among Canadian adults using CCHS 2004 cycle 2.2. The results of this study indicated that there is a U-shape relationship between the intake of carbohydrates and BMI [43]. Merchant and others (2009) showed that carbohydrate intakes of 290 to 310 g per day is associated with the lowest risk of obesity, while any level of carbohydrates intake above and below this range is associated with an increase in the likelihood of being overweight and obese. Specifically, the probability of being obese or overweight is significantly higher among those adults consuming the lowest level of carbohydrates (i.e., 179 g/day). In our analyses, the mean carbohydrate intakes were lowest in the ‘minimal grain’ pattern and in those with above 80% whole grain intakes (142.7 g/day and 194 g/day, respectively). In the study of Merchant et al. (2009), the average carbohydrate intakes of about 197 g/day was associated with an average BMI of around 27. Therefore, the high proportion of obese and overweight adults in the highest WGCs and ‘minimal grain’ consumption pattern might be explained by a considerably lower intake of carbohydrates.

The energy intakes across the highest WGCs and ‘minimal grain’ consumption pattern was significantly lower than in the balanced intake of grains. In addition, the intake of carbohydrates in the ‘lowest’, ‘low’, and ‘high’ WGCs were not significantly different from the balanced intake of GBFs. Meanwhile, the mean intake of energy decreased with an increase in the share of whole grains in the total grain-intake. Therefore, these observations raise the questions of whether other factors, along with carbohydrates and energy consumption, could affect the prevalence of obesity across the patterns of whole-grain consumptions.

**Figure 1:**
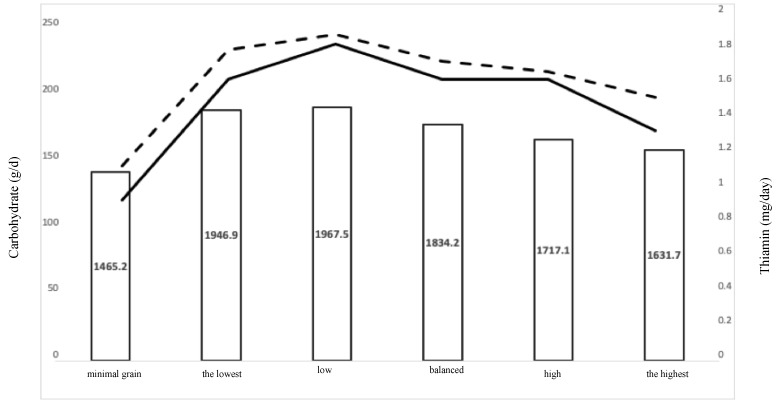
The intakes of energy, carbohydrates, and thiamin across WGCs. The bars indicate energy intake. Solid and dashed lines represent carbohydrate and thiamin intakes, respectively.

One possible factor is the thiamin intake. Intakes of thiamin across patterns of whole-grain consumption follow the same trend as the decrease in carbohydrates. Figure 1 shows the intake of carbohydrates (dashed line), thiamin (solid line), and energy across the patterns of whole grains consumption. Thiamin is known for its role in catalyzing glucose metabolism [44]. Studies have shown that the lower intakes of thiamin are associated with a higher frequency of obesity and overweight [44,45]. Therefore, the lower percentage of obese and overweight adults in the balanced intakes of grain might be explained by the balanced intake of carbohydrates, energy, and thiamin in this group. The importance of thiamin in the metabolism of glucose, and consequently its association with obesity, is indicated in a systematic review [45]. However, a study indicated that the prevalence of diabetes and obesity in the U.S were associated with higher intakes from the vitamin B family, such as niacin and thiamin, during the last 50 years. In addition, the impact of other factors, such as intake of other food groups, physical activity, SES, and lifestyle factors, should not be ignored in lower levels of obesity and overweight among adults with a balanced intake of grain products. In this case, we controlled for several factors in the logistic regression analyses, and the association between a balanced intake of grains and the probability of being obese or overweight were confirmed. However, future studies could examine other factors explaining the lower prevalence of obese and overweight adults within the balanced intake of grains group.

This study has some limitations. The dietary intake data in CCHS, are based on 24 h recalls, that are likely to be subject to over or under-reporting [46]. Therefore, the assessment of the nutrient intakes from food sources used in this study could have errors, although we excluded the implausible intakes reporting less than 200 and above 8,000 kcal/day. Associations between intakes of carbohydrates, thiamin, and energy with the frequency of obesity need to be examined in future studies. Furthermore, in this study we defined the patterns of whole-grain consumption based on the share of whole-grain consumption from the total daily intake of GBFs. Therefore, the results should be interpreted with caution. Specifically, a study by Ross et al. (2015) suggests, upon examining the health impact of GBFs intakes, that the number of servings or the weight of GBFs consumed should be used as the unit assessment [47]. It should also be mentioned that the data used in this study was confidential, and the sample sizes were only released in the form of weighted frequencies.

## 5. Conclusions

The results of our analyses using PSM and comparison of mean intakes of diet quality and intakes of some key nutrients in children, adolescents, and adults in Canada showed higher intakes of whole grain, beyond the balanced intake (between 40% to 60% whole grain), do not necessarily lead to optimal nutrient intakes. The intakes of calcium, iron, and especially folate was found to significantly decrease as the share of the whole-grain intakes in the total grain-intake increased In other words, this study indicated that the relationship between the share of whole grain in the intakes of diet and diet quality has a non-linear nature. Furthermore, the percentage of obese and overweight adults with balanced intakes was significantly lower in comparison with all other patterns of grain intakes. Balanced intakes of grains were more likely to be associated with lower risks of overweight and obesity. 

## Figures and Tables

**Table 1:** The percentage of Canadian children and adolescents (2- to 18-years-old) and adults (19-years-old and above) across whole-grain consumption patterns.

	Children and Adolescents (%) (n = 6,395,152) *	Adults (%) (n = 27,061,760) *
**The Lowest (WGCs: 0% to 20%)**	63.8	59.2
**Low (WGCs: 20% to 40%)**	15.6	12.4
**Balanced (WGCs: 40% to 60%)**	8.5	8.8
**High (WGCs: 60% to 80%)**	5.1	5.4
**The Highest (WGCs** **≥ 80%)**	3.4	7.3
**‘Minimal grain’ Consumers (total grain intake < 1 serving/day)**	3.6	6.2

WGCs—the share of whole grain intakes from the total daily grain intake. * n = population size.

**Table 2:** Socioeconomic status, body weight, and lifestyle factors of adults (19-years-old and above) across whole-grain consumption patterns.

	‘Minimal grain’ Consumers (Total Grain Intake <1 Serving/Day)	The Lowest (WGCs: 0% to 20%)	Low (WGCs: 20% to 40%)	Balanced (WGCs: 40% to 60%)	High (WGCs: 60% to 80%)	The Highest (WGCs: ≥80%)
Age, mean years (Mean, SEM)	47.9 (0.99)	48.13 (0.27)	49.4 ^†^ (0.85)	51.6 (0.9)	54.5 ^†^ (1.05)	53.8 (0.95)
Male (%, SE)	40.2 (2.89)	51.2 ^†^ (0.57)	53.8 ^†^ (1.93)	45.3 (2.23)	50.8 (2.69)	44.8 (2.71)
White (%, SE)	76.0 (2.94)	75.9 (0.92)	75.0 (2.02)	73.1 (2.38)	69.3 (2.87)	71.7 (2.7)
Education (% university grad, SE)	37.8 (2.92)	36.8 (0.99)	42.2 ^†^ (2.08)	36.4 (1.95)	50.5 ^†^ (3.1)	40.6 (2.8)
Food Secure (%, SE)	86.7 (2.02)	87.4 ^†^ (0.67)	91.8 (1.43)	91.2 (1.81)	90.6 (2.13)	89.7 (1.76)
Immigrant (%, SE)	26.1 (2.96)	25.8 (0.92)	27.9 (1.82)	29.6 (2.22)	37.1 ^†^ (2.69)	30.8 (2.69)
Married (%, SE)	57.6 ^†^ (2.8)	63.7 (0.90)	63.8 (2.10)	65.5 (2.35)	68.7 (2.8)	67.3 (2.4)
Urban Residents (%, SE)	79.9 (2.9)	81.4 (0.82)	85.9 (1.62)	83.4 (2.07)	89.8 ^†^ (2.1)	80.7 (2.5)
Obesity (% SE)	60.8 ^†^ (3.8)	62.1 ^†^ (1.3)	60.5 ^†^ (3)	54.6 (3.5)	69.4 ^†^ (3.7)	64.8 ^†^ (3.5)
BMI (Mean, SEM)	27.32 (0.49)	27.44 ^†^ (0.15)	26.62 (0.31)	26.58 (0.38)	27.72 ^†^ (0.45)	27.75 ^†^ (0.44)
Smoker (%, SE)	25.7 ^†^ (2.97)	21.6 ^†^ (0.89)	13.1 (1.41)	11.9 (2.1)	11.8 (1.84)	11.1 (1.64)
Active (% individual with at least 150 min of physical activity per week)	64.5 ^†^ (3.6)	71 (1.1)	77.3 (1.7)	75.5 (2.3)	75.6 (2.6)	73.1 (2.7)
Supplement consumer (% individual who took any supplement) (%, SE)	46.8 ^†^ (2.88)	42.7 ^†^ (0.84)	50.7 ^†^ (1.96)	57.4 (2.41)	60.1 (2.86)	51.7 ^†^ (2.8)

^†^ Significantly different from the balanced intake of grain. WGCs refers to the share of whole grain intakes from the total daily grain intake. SEM—standard error of the mean. SE—standard error. BMI—body mass index.

**Table 3:** Socioeconomic status, body weight, and lifestyle factors of children and adolescents (2- to 18-years-old) across whole-grain consumption patterns.

	‘Minimal grain’ Consumers (Total Grain Intake <1 Serving/Day)	The Lowest (WGCs: 0% to 20%)	Low (WGCs: 20% to 40%)	Balanced (WGCs: 40% to 60%)	High (WGCs: 60% to 80%)	The Highest (WGCs: ≥80%)
Age (mean, SEM)	9.93 (0.5)	10.12 ^†^ (0.09)	9.62 (0.23)	9.55 (0.36)	9.24 (0.51)	9.09 (0.54)
Male (%, SE)	48.7 (0.84)	50.2 ^†^ (0.27)	48.5 (0.85)	55.4 (0.96)	42.8 ^†^ (1.08)	50.3 (0.78)
White (%, SE)	63.1 (0.95)	67.1 (0.37)	67.4 (0.95)	69.6 (1.01)	70.9 (1.57)	68.9 (0.93)
Education (% university grad, SE)	44.1 (0.83)	41.9 ^†^ (0.32)	46.7 (0.86)	52.3 (0.96)	53.08 (1.29)	51.7 (0.78)
Food security (%, SE)	81.0 (1.07)	83.2 ^†^ (0.34)	85.6 (1.10)	87.4 (1.21)	84.0 (1.54)	85.5 (0.97)
Immigrant (%, SE)	9.4 (0.32)	8.4 ^†^ (0.15)	9.2 (0.37)	12.9 (0.52)	8.1 (0.55)	14.0 (0.45)
Urban Residents (%, SE)	77 (1.03)	82.6 (0.34)	83.3 (1.12)	82.9 (1.26)	75.2 (1.46)	82.9 (0.98)
BMI Z-score (mean, SEM)	0.75 (0.19)	0.48 (0.05)	0.32 (0.1)	0.51 (0.1)	0.4 (0.14)	0.43 (0.23)

^†^ Significantly different from the balanced intake of grain. WGC—the share of whole grain intakes from the total daily grain intake. SEM—standard error of the mean. SE—standard error.

**Table 4:** Average treatment effects on the treated and mean values of diet quality, energy, and nutrients intake of adults (19-years-old) across WGCs.

		NRF	Energy (Kcal/d)	Folate ^a^ (DFE/d)	Iron ^a^ (mcg/d)	Calcium ^b^ (mg/d)	Fiber (g/d)	Magnesium ^b^ (mg/d)	Thiamin ^a^ (mg/d)	Carbohydrates (g/d)	Riboflavin ^a^ (mg/d)	Niacin ^a^ (mg/d)	Potassium (mg/d)
**‘Minimal grain’ Consumers (total grain intake<1 serving/day)**	**ATE (SE)**	−9.5 *^,†^ (−1.4)	−513.2 *^,†^ (−14.8)	−183.1 *^,†^ (−16.4)	−4.6*^,†^ (−16)	−214 *^,†^ (−10)	−4.9 *^,†^ (−11.1)	−54.7 *^,†^ (−7.6)	−0.7 *^,†^ (−17.9)	−83 *^,†^ (−19.7)	−0.4 *^,†^ (−8.2)	−7.6 *^,†^ (−8)	−232.7 *^,†^ (−3.9)
	**Mean (SEM)**	515.9 (11.3)	1465.2 (76.8)	265.1 (14.7)	8.3 (0.4)	582.4 (31.4)	12.4 (0.6)	264.1 (10.8)	0.9 (0)	142.7 (2.2)	1.6 (0.1)	34.6 (2.3)	2540 (83.2)
**The Lowest (WGCs: 0% to 20%)**	**ATE (SEM)**	−58.1 *^,†^ (−18.8)	159.1 *^,†^ (9)	109.2 *^,†^ (21)	0.5 *^,†^ (3.6)	−5.6 ^†^ (−0.5)	−3.4 *^,†^ (−14.7)	−39.8 *^,†^ (−11.3)	0.1 *^,†^ (6.9)	17.2 *^,†^ (7.7)	0.1 *^,†^ (6.2)	1.9 *^,†^ (4)	−139.1*^,†^ (−5.1)
	**Mean (SEM)**	491.13 (2.72)	1946.9 (19.2)	483.6 (6.1)	12.6 (0.1)	794 (11.7)	15.7 (0.2)	293.7 (3.4)	1.6 (0)	230.1 (2.2)	2 (0)	40.5 (0.5)	2649.3 (26.8)
**Low (WGCs: 20% to 40%)**	**ATE (SE)**	32.2 *^,†^ (7.3)	90.6 (3.3)	25.9 *^,†^ (3.2)	1.6 *^,†^ (7.1)	102.8 * (6.1)	2.6 *^,†^ (8)	33 *^,†^ (6.4)	0.2 *^,†^ (6.1)	20.5 *^,†^ (6.1)	0.1 *^,†^ (3.6)	1.4 * (2.1)	134 * (3.3)
	**Mean (SEM)**	555.02 (5.01)	1967.5 (38.6)	458.1 (9.6)	13.7 (0.3)	836.6 (19.6)	19.7 (0.4)	339.3 (5.8)	1.8 (0.1)	241.8 (4.8)	2(0)	40.5 (1)	2841.7 (50.7)
**Balanced (WGCs: 40% to 60%)**	**ATE (SE)**	48.8 * (9.9)	39.9 (1.4)	−33.5 * (−3.5)	1 * (3.8)	69.2 * (3.6)	4 (10.3)	54.8 * (8.9)	0.1 * (1.9)	11.7 * (3.2)	−0.01 * (−0.2)	0.9 (1.1)	245.7 * (5.2)
	**Mean (SEM)**	565.25 (7.32)	1834.2 (44)	397.1 (10.4)	12.6 (0.3)	855.9 (36.2)	20.5 (0.6)	342.8 (7.6)	1.6 (0.1)	221.6 (5.5)	1.8 (0.1)	37.3 (0.9)	2789.8 (60.6)
**High (WGCs: 60% to 80%)**	**ATE (SE)**	43.6 * (6.8)	−42.4 (−1.2)	−94.9 *^,†^ (−9)	−0.3 † (−1.1)	22.9 ^†^ (1)	3.8 (6.7)	46.9 * (6.3)	−0.1 *^,†^ (−2.2)	−6 *^,†^ (−1.3)	−0.1 (−1.3)	0.4 (0.4)	130.5 * (2.1)
	**Mean (SEM)**	575.57 (6.81)	1717.1 (41.6)	352.4 (12.7)	11.9 (0.4)	736.9 (26)	22.1 (0.7)	348.1 (9.7)	1.6 (0.1)	213.3 (5.2)	1.7 (0)	36.4 (1.3)	2715.8 (77.9)
**The Highest (WGCs:** **≥ 80%** **)**	**ATE (SE)**	51.7 * (9.4)	−175.3 *^,†^ (−5.4)	−118 *^,†^ (−11.9)	−0.8 *^,†^ (−2.9)	−37.3 *^,†^ (−1.7)	3.8 (8.1)	40 * (5.8)	−0.2 *^,†^ (−5.9)	−18.9 *^,†^ (−4.7)	−0.2 *^,†^ (−4.4)	−2.9 *^,†^ (−3.4)	119.6 * (2.3)
	**Mean (SEM)**	576.2 (7.59)	1631.7 (39.4)	307.9 (11.9)	11.1 (0.4)	693.8 (24.1)	20.5 (0.6)	332.9 (9.2)	1.3 (0)	194 (5.6)	1.7 (0.1)	35.7 (1.2)	2712 (75.5)

* Significantly different from zero (*p* < 0.05). ^†^ Significantly different from the balanced intake of grain. ATE—Average treatment effects on the treated. WGC—the share of whole grain intakes from the total daily grain intake. SEM—standard error of the mean. ^a^ Nutrient that was added for the mandatory fortification of refined flour. ^b^ Nutrient that was added for voluntary fortification of refined flour. NRF: nutrient rich food index 9.3. DFE: dietary **folate** equivalent.

**Table 5:** Average treatment effects on the treated and mean values of diet quality, energy and nutrients intake in children and adolescents (2- to 18-years-old).

		Energy (Kcal/d)	NRF	Folate ^a^ (DFE/d)	Iron ^a^ (mcg/d)	Calcium ^b^ (mg/d)	Fiber (g/d)	Magnesium ^b^ (mg/d)	Thiamin ^a^ (mg/d)	Carbohydrates (g/d)	Riboflavin ^a^ (mg/d)	Niacin ^a^ (mg/d)	Potassium (mg/d)
**'** **Minimal grain’ Consumers (total grain intake < 1 serving/day)**	**ATE (SE)**	−563 *^,†^ (51.6)	−32.7 *^,†^ (10.4)	−200 *^,†^ (14.3)	−5.1 *^,†^ (0.4)	−327.3 *^,†^ (40.3)	−4.8 * (0.5)	−76.9 *^,†^ (9)	−0.7 *^,†^ (0.1)	−88 *^,†^ (6.8)	−0.5 *^,†^ (0.1)	−9.9 *^,†^ (1.4)	−313.8 *^,†^ (86.6)
	**Mean (SEM)**	1252 (61.6)	463 (11.8)	160.3 (9.4)	10.2 (0.5)	651.6 (50.6)	9.6 (0.5)	174.8 (9.1)	0.9 (0.1)	154.2 (9.1)	1.3 (0.1)	22.6 (1.6)	1987.5 (120.2)
**The Lowest (WGCs: 0% to 20%)**	**ATE (SE)**	150.4 *^,†^ (24.4)	−44.4 *^,†^ (3.8)	116.5 *^,†^ (6.9)	0.4 (0.2)	−3.3 (18.4)	−2.6 *^,†^ (0.3)	−26.6 *^,†^ (3.9)	0.1 *^,†^ (0)	19 *^,†^ (3.4)	0.2 *^,†^ (0)	2.1 *^,†^ (0.6)	−77 *^,†^ (36.3)
	**Mean (SEM)**	1884.5 (19.6)	496.1 (2.9)	322.3 (4.3)	6.8 (0.4)	944 (15)	14.3 (0.2)	248.2 (3)	1.6 (0)	250.5 (2.7)	1.9 (0)	34.7 (0.5)	2381.9 (26.1)
**Low (WGCs: 20% to 40%)**	**ATE (SE)**	−4.7 (30.4)	34.8 * (4.8)	−10.1 *^,†^ (9.5)	1.2 *^,†^ (0.3)	64 * (22.8)	2.1 * (0.3)	20.6 *^,†^ (4.7)	0.1 *^,†^ (0)	7.3 ^†^ (4.2)	0 (0)	−0.6 (0.7)	85.8 (45.8)
	**Mean (SEM)**	1776.3 (35)	549.6 (5.3)	285.3 (6.4)	12.2 (0.1)	973.9 (23.1)	16.5 (0.4)	269.8 (5.2)	1.6 (0)	245.5 (5.1)	1.8 (0)	32.3 (0.8)	2403.1 (51.9)
**Balanced (WGCs: 40% to 60%)**	**ATE (SE)**	−31.4 (36.9)	33.4 * (6)	−80.1 * (11.7)	0 (0.4)	63.9 * (31.9)	2.3 * (0.5)	37.1 * (6.5)	−0.1 * (0.1)	−5.4 (5)	0 (0.1)	0.7 (1)	53 (58)
	**Mean (SEM)**	1796.7 (49)	564.3 (8.4)	289.5 (16.2)	12.8 (0.3)	1044.1 (48.2)	18 (0.6)	307.9 (12.8)	1.5 (0.1)	240.8 (6.2)	1.9 (0.1)	35.3 (2.2)	2553.7 (87.1)
**High (WGCs: 60% to 80%)**	**ATE (SE)**	−27 (50.1)	49 * (8.6)	−91.2 * (13.8)	0.6 (0.5)	26.1 (37.5)	5.1 *^,†^ (0.7)	51.8 *^,†^ (8.1)	0 (0.1)	3.5 (7.2)	−0.1 (0.1)	0.1 (1.2)	211.4 *^,†^ (74.7)
	**Mean (SEM)**	1649.7 (69.2)	555.1 (9.1)	237 (13.3)	13.2 (0.7)	940.9 (52.9)	18.2 (0.9)	271.2 (10.8)	1.4 (0.1)	230.8 (10.1)	1.6 (0.1)	28.8 (1.4)	2307.1 (93.2)
**The Highest (WGCs:** **≥ 80%** **)**	**ATE (SE)**	−205 *^,†^ (49.5)	54 * (8.8)	−175.3 *^,†^ (15.8)	−1.7 *^,†^ (0.4)	−29.9 (45.8)	3.4 *^,†^ (0.7)	31* (9.3)	−0.2 *^,†^ (0.1)	−31.3 *^,†^ (7)	−0.2 * (0.1)	−1.8 (1.2)	130.9 (85.8)
	**Mean (SEM)**	1678.9 (81.7)	567.3 (11.8)	223.5 (13.8)	11.3 (0.9)	926.2 (47.4)	18.2 (0.8)	291.4 (11.5)	1.3 (0.1)	218.3 (7.8)	1.7 (0.1)	32.1 (1.7)	2650.4 (174.9)

* Significantly different from zero (*p* < 0. 05). ^†^ Significantly different from the balanced intake of grain. WGC—the share of whole grain intakes from the total daily grain intake. SEM—standard error of the mean. ^a^ Nutrient that is added in the mandatory fortification of refined flour. ^b^ Nutrient that is added in the voluntary fortification of refined flour.

**Table 6:** Adjusted odd ratios showing factors associated with obesity and overweight.

Variable		Odd Ratio (Standard Errors)	95% Confidence Interval
**Whole-Grain Consumption Patterns**	The Lowest (0% to 20%)	1.48 ** (0.24)	(1.09–2.03)
	Low (20% to 40%)	1.33 (0.27)	(0.89–1.99)
	Balanced (40% to 60%)	-	-
	High (40% to 60%)	1.78 ** (0.43)	(1.1–2.86)
	The Highest (≥80%)	1.65 ** (0.33)	(1.11–2.44)
	Minimal grain Consumers	1.55 * (0.38)	(0.95–2.52)
**White**		1.36 ** (0.18)	(1.04–1.77)
**Higher Education in Household**		0.82 ** (0.08)	(0.67–1)
**Immigration Status**		0.82 * (0.1)	(0.64–1.03)
**Smoker**		0.67 *** (0.08)	(0.52–0.86)
**Food Secure**		0.72 ** (0.12)	(0.52–0.99)
**Age**		1.14 *** (0.02)	(1.11–1.18)
**Age-Squared**		1 *** (0.0)	(1–1)
**Male**		1.99 *** (0.19)	(1.65–2.42)
**Constant**		0.06 ** (0.03)	(0.02–0.14)

The results are reported from logistic regression analyses where in the case of patterns of whole-grain consumption, the balanced intake of grains was used as the reference group. In the case of SES and health-related variables, only the variables that were significantly different from zero are reported. *significantly different from zero at the 10% level. **significantly different from zero at the 5% level. ***significantly different from zero at the 1% level.
